# Prone Positioning May Improve the Treatment of Diffuse Alveolar Hemorrhage and Severe Acute Respiratory Distress Syndrome (ARDS) Secondary to ANCA Associated Vasculitis: A Case Report

**DOI:** 10.3390/life12020235

**Published:** 2022-02-03

**Authors:** Shang-Ju Wu, Yong-Chen Hsu, Kao-Lun Wang, Pin-Kuei Fu

**Affiliations:** 1 Department of Internal Medicine, Taichung Veterans General Hospital, Taichung 40705, Taiwan; alanwu0206@hotmail.com; 2 Department of Pathology and Laboratory Medicine, Taichung Veterans General Hospital, Taichung 40705, Taiwan; shuyongjen@hotmail.com; 3 Department of Radiology, Taichung Veterans General Hospital, Taichung 40705, Taiwan; y0107124@yahoo.com.tw; 4 Department of Critical Care Medicine, Taichung Veterans General Hospital, Taichung 40705, Taiwan

**Keywords:** prone positioning (PP), diffuse alveolar hemorrhage (DAH), acute respiratory distress syndrome (ARDS), ANCA-associated vasculitis (AAV)

## Abstract

Diffuse alveolar hemorrhage (DAH) secondary to anti-neutrophil cytoplasmic antibodies (ANCA)-associated vasculitis is rare in clinical practice and may present as severe acute respiratory distress syndrome (ARDS) with high mortality. Extracorporeal membrane oxygenation (ECMO) has been reported to be a salvage treatment providing the time necessary for immunosuppressive treatment in cases accompanied by severe ARDS. Prone positioning (PP) has been proven to reduce the mortality in patients with severe ARDS. However, there is no consensus about choosing PP or ECMO in severe ARDS due to DAH secondary to ANCA-associated vasculitis. We reported a case of microscopic polyangiitis (MPA)-related DAH and severe ARDS treated with PP successfully providing the time necessary for early glucocorticoids and plasma exchange to control the underlying disease. Since anticoagulation therapy is not necessary in PP, it does not increase the risk of bleeding tendency unlike ECMO. PP has a life-saving role in the management of patients with severe ARDS due to ANCA-associated pulmonary vasculitis.

## 1. Introduction

ARDS, a life-threatening condition, causes severe mortality that varies from 34.9% for mild ARDS to 46.1% for severe ARDS, even with mechanical ventilation or even ECMO support [1]. DAH, a rare cause of ARDS, presents with hemoptysis resulting from intra-alveolar RBC accumulation and may hinder alveolar oxygenation and progress to hypoxia [2]. Here, we report a case of a 74-year-old male who was diagnosed with DAH-related ARDS treated successfully with prone positioning. Serology tests proved it to be ANCA-associated vasculitis [3]. We also reviewed the related literature and proposed the preferable choice of prone positioning or ECMO under such circumstances.

## 2. Case Presentation

**Figure 1:**
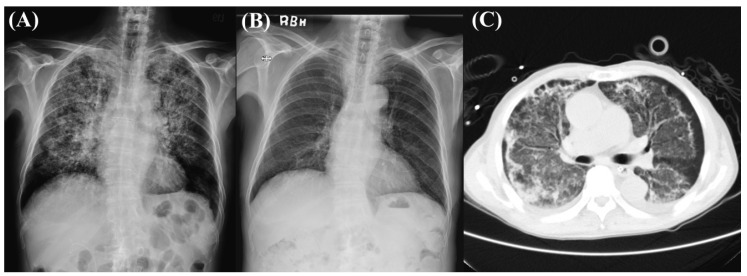
(**A**) Chest X-ray (CXR) before intubation: bilateral asymmetric patches of hazy opacity, relatively sparing lateral lung bases. (**B**) CXR two weeks after extubation: great resolution of bilateral infiltration and relatively clear lung fields. (**C**) Chest computed tomography showed diffuse ground glass opacities mixed with patchy consolidation, predominantly in upper and middle lung zones, with subpleural sparing.

A 74-year-old male with chronic ureteral stricture was admitted with a urinary tract infection (UTI). There were no respiratory symptoms initially. However, he had persistent spiking fever for over one week and received adequate and effective antibiotic treatment for UTI. On the 6th day after admission, he started to have symptoms of coughing with blood-tinged sputum. An episode of acute massive hemoptysis followed by hypoxemic respiratory failure developed on the 9th day of admission. Laboratory studies showed a marked decrease in hemoglobin from 12.3 to 8.4 g/dL within 1 day. Before intubation, arterial blood gas data were as follows: pH 7.332, HCO_3_ 25.3 mmol/L, carbon dioxide pressure 48.9 mmHg, and oxygen partial pressure 118.6 mmHg supplemented with 100% fraction of inspired O_2_ (FiO_2_). Thoracic radiography revealed bilateral asymmetric patches with hazy opacity and relative sparing of the lateral lung bases (Figure 1A).

**Figure 2:**
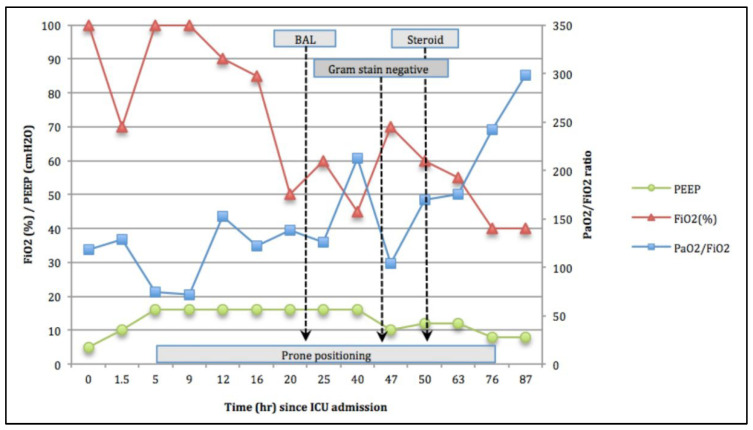
PF ratio before, during, and after prone position. BAL: bronchoalveolar lavage, PEEP: positive end expiratory pressure. PF ratio: PaO_2_/FiO_2_.

He was intubated urgently and was then transferred to the intensive care unit (ICU) under critical condition. Intravenous and inhaled tranexamic acid accompanied by fresh frozen plasma transfusion were administered immediately at the ICU. As the PaO_2_/FiO_2_ (P/F ratio) of this patient was 74.6 and he presented with patches over the bilateral lung field within one week, he was diagnosed with severe ARDS, in accordance with the Berlin definition published in 2012 [4]. Prone positioning (PP) and extracorporeal membrane oxygenation (ECMO) are both used as rescue therapies for severe ARDS [4]. In view of the active bleeding, which is a contraindication for ECMO, and the advantage of posture drainage that PP provides, we chose PP at the 5th hour after ICU admission as a salvage therapy for this patient, who presented with massive pulmonary hemorrhage and severe ARDS. Under prolonged PP treatment (continuous PP treatment for at least 72 h), his P/F ratio improved steadily over the 12 h period following admission to the ICU (Figure 2).

**Figure 3:**
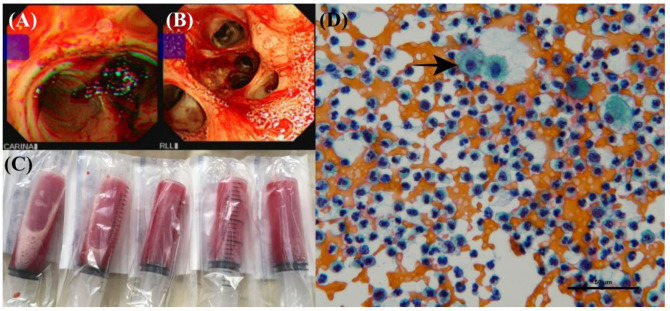
(**A**) Diffuse alveolar hemorrhage (DAH) in BAL (bronchoalveolar lavage), at carina. (**B**) DAH in BAL, at right lower lung (RLL). (**C**) Bloody specimen from BAL fluid. (**D**) BAL fluid microscopically filled with bloody content and a hemosiderin-laden macrophage inside (arrow).

On the 2nd ICU Day, we performed a bronchoscopy exam and bronchoalveolar lavage (BAL) to check for bleeding and to survey the BAL fluid. With grossly bright red (Figure 3A–C) and microscopically bloody content with no organisms observed (Figure 3D) in the BAL fluid, the findings were compatible with diffuse alveolar hemorrhage. Additionally, a large volume of watery bloody sputum was drained out of the endotracheal tube in the first two days after PP treatment. Pulmonary-renal syndrome was suspected on the second ICU day due to massive pulmonary hemorrhage accompanied by microscopic hematuria, which was revealed by a routine urine exam. Vasculitis survey, including the tests for anti-neutrophil cytoplasmic antibodies (ANCA) and anti–glomerular basement membrane (anti-GBM) antibody, was arranged after the BAL exam. Methylprednisolone 40 mg per day was administered on the third ICU day due to suspicion of vasculitis-related diffused pulmonary hemorrhage (DPH). The pulmonary hemorrhage decreased in volume and the P/F ratio improved to 174 at the 72nd hour after PP treatment.

On the fourth ICU Day, prone positioning was ended and the patient was returned to the supine position (Figure 2). The patient was in a relatively stable condition so we arranged chest computed tomography (CT), which revealed diffuse ground glass opacities mixed with patchy consolidation, predominantly in the upper and middle lung zones with subpleural sparing, compatible with the presentation of pulmonary hemorrhage (Figure 1C). He was extubated successfully on the seventh ICU Day. The results of the vasculitis survey were also obtained on the seventh ICU Day and proved that DAH was caused by MPA (myeloperoxidase level: 91 IU/mL). We started plasma exchange in addition to systemic steroid treatment for five days on the seven ICU Day. He was discharged without complications on the 23rd hospital day. CXR performed two weeks after extubation revealed considerable resolution of bilateral infiltration (Figure 1B).

## 3. Discussion

Antineutrophil cytoplasmic antibody (ANCA) disease is characterized by small-vessel vasculitis and may manifest as microscopic polyangiitis (MPA), granulomatosis with polyangiitis, and eosinophilic granulomatosis with polyangiitis [5]. Systemic involvement is common, including the kidneys, lung, skin, and neuropathy [3]. Diffuse alveolar hemorrhage (DAH) is a clinicopathologic syndrome characterized by intra-alveolar RBC accumulation and etiologies based on histology include pulmonary capillaritis, bland pulmonary hemorrhage, and diffuse alveolar damage [6]. Of note, ANCA-associated vasculitis is the most common form of pulmonary capilllaritis [6]. A previous study demonstrated that the most common cause for ICU admission was active vasculitis presented with massive pulmonary hemorrhage and higher in-ICU mortality [7].

The management of DAH depends on the specific etiology that is involved. For patients who develop massive pulmonary hemorrhage accompanied by acute respiratory failure, ventilatory support for severe hypoxemia is mandatory in the critical status [8]. Other therapies, such as activated factor VII [9,10] and prone positioning [11,12], are less well studied and thus it is not known how effective they are. ECMO has been shown to be effective in the treatment of hypoxemic DAH based on literature reviews [13,14,15,16], but the use of anticoagulation agents may exacerbate bleeding [17]. Veno-venous (VV)-ECMO support was indicated in the condition of refractory hypoxemia or uncontrollable hypercapnia accompanied by respiratory acidosis (pH < 7.2) despite optimized ventilator settings according to previous research [18]. Owing to the possible risks of VV-ECMO and the reported advantages of prone positioning (Table 1) [16,17,18,19,20,21,22,23,24], we decided to use prone positioning to treat the hypoxemia. The protocol for PP therapy in our RICU was at least 48 to 72 h of continuous therapy as described in our previous studies [25]. Prone positioning has several beneficial effects with respect to ventilation [26]. It helps increase transpulmonary pressure and reduces the pleural pressure gradient from the nondependent to dependent regions, thereby achieving more homogenous lung aeration [26]. It also decreases shunting when in the prone position [26]. The use of PP in patients with ARDS, along with high positive end expiratory pressure (PEEP), helps to minimize barotrauma and atelectrauma, which also brings benefits to patients with COIVD-19 infection [27]. In patients who have a diffused pulmonary hemorrhage, the traditional supine position tends to result in blood remaining in the posterior part of the lung through gravity, which worsens ventilation and perfusion (V/Q) mismatch, however, PP could minimize the V/Q mismatch and improve the oxygenation of patients. Furthermore, PP helps the postural drainage of pulmonary hemorrhage through both the alveoli and large airways. Moreover, it is very difficult to deal with patients who receive both PP therapy and ECMO at the same time. Based on this successful experience, we recommend prone positioning in certain cases with pulmonary hemorrhage as it may shorten the hypoxemia course.

## 4. Conclusions

ARDS is a life-threatening condition. DAH-associated ARDS is rare in clinical practice. Prone positioning in DAH-associated ARDS appeared to help reverse hypoxemia by minimizing the V/Q mismatch and improving the oxygenation of our patient. We suggest that prone positioning may have a positive effect in terms of improving oxygenation and is superior to ECMO in the management of DAH-associated ARDS for reducing the risk of mortality and complications of bleeding.

## Figures and Tables

**Table 1:** Comparison of veno-venous extracorporeal membrane oxygenation (VV-ECMO) and prone positioning. ARDS: acute respiratory distress syndrome.

Characteristics	Veno-Venous ECMO (VV-ECMO)	Prone Positioning
Indications	Very severe ARDS; Bridge to lung transplantation; Primary graft dysfunction after lung transplantation.	Severe ARDS regardless of etiologies
Contraindications	Absolute Contraindications: Irreversible underlying process when the patient is not a candidate for lung transplantation; Cardiogenic failure and severe chronic pulmonary hypertension (mean pulmonary artery pressure >50 mmHg). Relative Contraindications: Contraindication for anticoagulation; Advanced age; Obesity.	Serious burns or open wounds on the ventral body surface; Spinal instability; Pelvic fractures; Life-threatening cardiac arrhythmias or hypotension.
Complications	Medical Complications: Bleeding (including intracranial hemorrhage, pulmonary and cannula bleeding); Pneumothorax; Deep vein thrombosis or pulmonary embolism; Cannula infection. Mechanical Complications Oxygenator failure; Cannula failure.	Pressure ulcers Endotracheal tube obstruction

## Data Availability

Not applicable.
